# Information-seeking process and clinical scenario solving: introduction of a new tool in nursing education

**DOI:** 10.1186/s12909-023-04943-5

**Published:** 2023-12-12

**Authors:** Hakimeh Vahedparast, Mahasty Ganjoo, Farkhondeh Ghasemi, Leila Dehghani

**Affiliations:** 1 School of Nursing and Midwifery, Bushehr University of Medical Sciences, Bushehr, Iran; 2 School of Paramedical Science, Bushehr University of Medical Sciences, Bushehr, Iran

**Keywords:** Concept Map, Information seeking process, Nursing education, Nursing student, Scenario-based learning, Search as Learning

## Abstract

**Background:**

Nursing education has recently undergone changes to improve care. These changes require innovative and transformative strategies in nursing education. Search as learning is one of the educational methods this study was conducted to determine the effect of the information searching process on scenario-based learning in nursing students.

**Methods:**

This study is a single group semi-experimental study that was conducted on 38 nursing students in 2021. Students first drew a concept map according to their existing knowledge about two scenarios (diabetes and trauma). The students then searched the medical databases and drew another concept map after the search. Data were analyzed using descriptive statistics, paired mean tests and Pearson correlation coefficients.

**Results:**

The results showed that the mean scores of the participants in the diabetes scenario before and after the search were 18.32 ± 5.50 and 19.13 ± 7.54, respectively, and those in the trauma scenario were 18.58 ± 7.69 and 29.61 ± 7, respectively (*P* < 0.01). The mean scores of the details of the conceptual map before and after the search in terms of themes, number of levels and relationships were statistically significant. In both scenarios, there was a positive correlation (*p* < 0.01) between learning and the number of correct webpages (r = 0.74 for trauma and r = 0.64 for diabetes), as well as between search time and the amount of learning (r = 0.77 for trauma and 0.64 for diabetes).

**Conclusions:**

The results showed that search as learning in nursing education scenarios led to student learning. It is recommended that nurse educators use this method as a tool in nursing education to increase students’ awareness and develop their thinking skills. Further research is recommended to determine the effectiveness of this method with other educational methods.

**Supplementary Information:**

The online version contains supplementary material available at 10.1186/s12909-023-04943-5.

## Background

Due to the increase in the content and complexity of nursing knowledge and the advancement of educational technology, the use of effective methods in the learning process has been considered. According to the WHO report, changes in nursing education are underway worldwide to improve health care, strengthen the nursing and midwifery workforce, and enhance the professional status of nurses. Therefore, innovative and transformative strategies in education are needed to meet the challenges of nursing education [1].

Today, the use of scenario-based learning is one of the new learning approaches in nursing education [2]. In this method, in addition to using their knowledge, students are trained in critical thinking, problem solving and creativity in a safe and realistic environment (scenarios). Many researchers believe that scenario-based learning is a type of active and dynamic learning and a learning strategy [3, 4]. In addition, many authors emphasize that this method strengthens students’ abilities and skills in analytical thinking, problem solving, communication and teamwork [5]. This improves self-reliance [6] and self-learning skills [7]. The available evidence suggests that the use of one teaching model, even a new model, is not enough to promote learning. A combination of teaching models should be used to stimulate active learning and thinking in learners [8–10].

Search as learning is one of the educational methods that has recently been evaluated by students as an effective and positive method [11]. The relationship between information seeking and learning can be divided into two categories: “learning to search” and “search as learning” [12, 13]. Search as learning means the formation of formative learning during the information search process [11]. This type of learning considers users’ behavior during the search process as an indicator of learning, tries to measure the amount of learning, and presents search as a learning strategy [14–16]. Search as learning leads to exploratory search. The basic theory of exploratory search is that the main function of the search system should not be to provide search results but to help users discover and overcome their uncertainty and to develop a kind of information-focused critical thinking that ultimately leads to intellectual growth and learning [16, 17]. This is also the main goal of the scenario-based learning approach. Marton [18]emphasizes that learning and information seeking have become closer due to the pedagogical ideas of constructivist theory, which is also the basis of the scenario-based learning approach, and the development of digital tools, which have changed the form and conditions of learning in modern society. XZhang aimed to determine user learning during the interactive search process with PubMed database data and concluded that the more documents stored and available in the databases, the greater the sense of learning in the searcher [19]. The results of a study by Bhatcharya and Guizda [20], using simple to complex scenarios, showed that reading the content (quickly browsing the text) led to more changes in participants’ verbal knowledge, but there were no significant differences in other search interactions, such as page views. Zhang and Changliu [21] also found that users’ prior knowledge played an important role in their search formula. This study also showed how prior knowledge and search results help users learn when searching for information. Chi [22] also investigated online health information searching, focusing on cancer patients and its effect on their health knowledge learning, and showed that the level of learning increased with searching.

The review of the literature shows that the topic of searching and its relationship with learning has been seriously considered by researchers for a long time and in parallel with the development of educational systems; however, the study of this topic has been neglected in the field of medical sciences, especially in nursing. In fact, using online search engines is one of the most common activities today. 81% of Americans rely heavily on information from the Internet when making important decisions [23]. Furthermore, a study in South African universities showed that 96.5% of nursing students use the Internet for academic purposes [24]; therefore, it is very important to investigate the fact that searching the Internet and information sources can lead to learning clinical topics. It should be emphasized that no research has been carried out in the area of scenario-based searching and learning and the interaction and close relationship between the two in the field of nursing, whereas in today’s educational world, the focus has shifted from teaching to learning [25].

### Objective and hypothesis

This research is a semi-experimental study with the aim of investigating the change in scenario-based learning through a use of search while learning in nursing students. From a practical point of view, if experimentally confirmed, the search process can be added to scenario-based learning strategies. This provides a combined method for training.

To conduct this research, students’ learning is measured before and after the intervention (search for learning) in two scenarios. Therefore, the first hypothesis of this research is:

#### Hypothesis 1

*There is a significant difference in the amount of learning in the students before and after searching for learning in the diabetes and trauma scenario.*

The amount of learning is also related to the two factors time and search result (the number of correct web pages found). (two sources) Therefore, this relationship was investigated in this research. The following hypothesis will be tested:

#### Hypothesis 2

*There is a significant relationship between the amount of learning and the number of correct web pages searched by students.*

#### Hypothesis 3

*There is a significant relationship between the amount of learning and the amount of time students spend searching.*

## Methods

### Study design

The current research was a semi-experimental one group pre-test post-test design and was conducted on nursing students of Bushehr University of Medical Sciences in 2021.

**Fig. 1:**
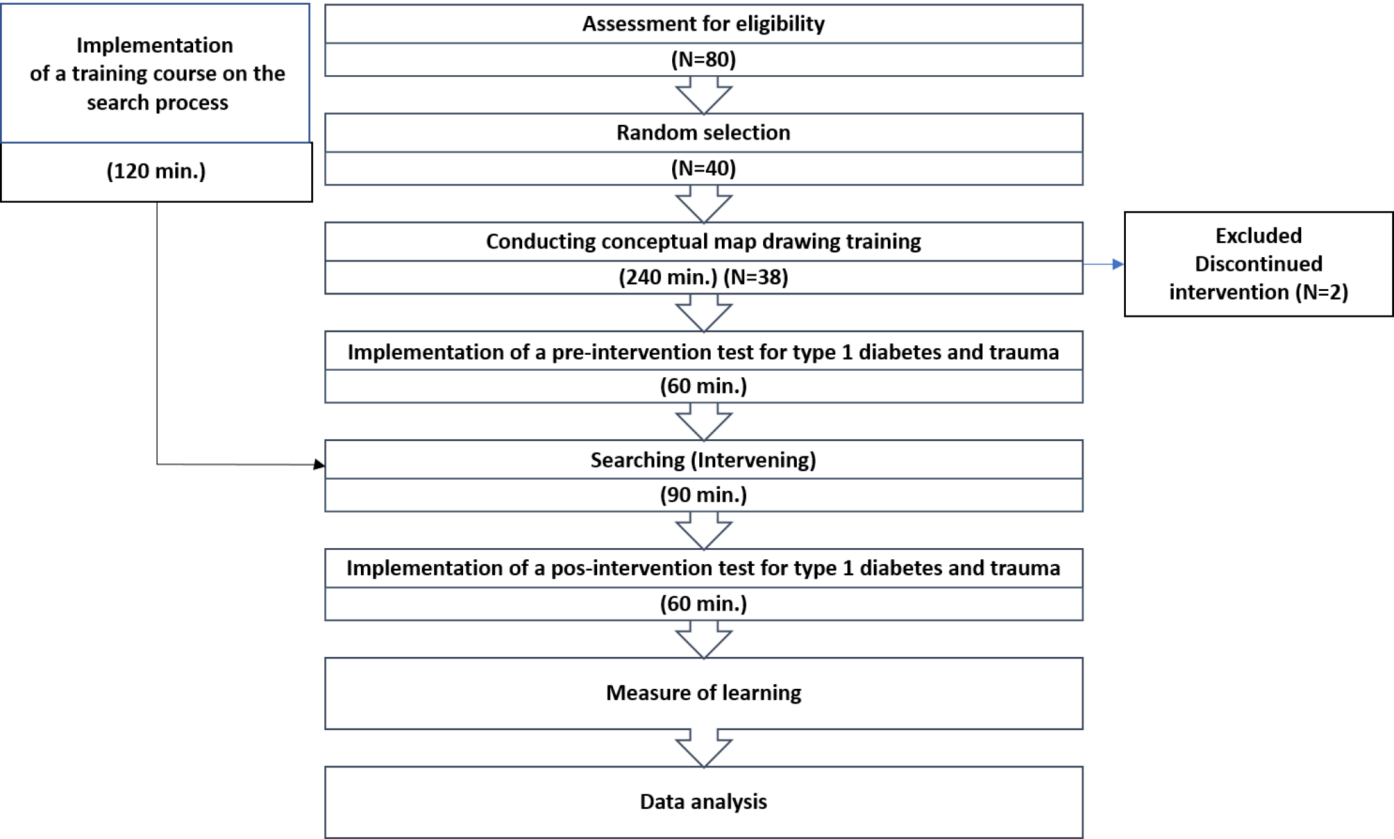
Study Design

After receiving ethical approval from the Ethics Committee of the University of Medical Sciences, the researcher started work, and according to the eligibility criteria, participants were randomly selected and enrolled in the study after obtaining informed consent. First, a briefing session was held to explain the objectives and process of the research. At this meeting, participants were assured that any information obtained from them would be kept strictly confidential. They were also told that they could withdraw from the study at any stage. Considering that the evaluation was done through concept maps, a 4-hour training workshop was held to familiarize the students with concept map drawing by a university lecturer with Master’s degree in nursing and PhD in medical education. Microsoft PowerPoint was used to present the content. During the first 60 min, an introduction was given to the definition of the concept map and its elements (concept, central concept, hierarchy, links, examples, linking words and extensions/complements). The types of concept maps (hierarchical, spider, flowchart and system) and their differences were then briefly introduced (30 min), but for the sake of equivalence, the focus of the training was on the hierarchical concept map, and a sample concept map was drawn for the students to familiarize themselves with (60 min). To solve the students’ problems in drawing a concept map, they were asked to draw a care plan and were given the necessary feedback (90 min). A workshop on the search process on the Internet was also held to unify students’ search skills. The instructor of the workshop was a member of the academic staff with a PhD in Library and Information Science, and to make the training more effective, the students were divided into two groups (19 people in each group). This workshop taught searching in Up-To-Date, Clinical-Key, PubMed and Google and Google Scholar search engines. The workshop took place in a room with independent stations equipped with computer systems connected to the Internet. Each student had a computer and searched the sites presented at the same time as the teacher, who also gave the necessary feedback. After the training, the test started the next day. The intervention process in the training took place after the pre-test. The amount of learning measured before and after the intervention was then calculated and used for evaluation and analysis. The following diagram (Fig. 1) provides a brief overview of the process of the study.

### Participants

Nursing students in the sixth semester of Bushehr University of Medical Sciences, who had completed the course of Information Technology in Nursing, were eligible to participate in the research, and 40 of them gave their informed consent to participate in all stages of the research. After the start of the study, two people were present only in the first stage and refused to cooperate in the second stage, so they were eliminated. A total of 38 students (17 females and 21 males) participated in the research. Inclusion criteria included 6th semester nursing, having passed the IT unit and medical-surgical courses [1–3] and Written informed consent to participate in the study. Failure to complete practice and assigned tasks were the exclusion criteria.

### Sample size calculation

Using the sample size formula for the paired group and the average effect size and taking into account a type І error of 0.05 and 90% power, approximately 32 samples were calculated, and the number of samples was determined taking into account 20% attrition. Of the 80 students who met the entry criteria, 38 were randomly selected. The samples were selected by lottery according to the number of students.

### Intervention

Prior to the intervention, two scenarios (management and care of patients with head trauma and type 1 diabetes) were presented to the students. These two scenarios were based on standard and international nursing bachelor’s textbooks. The students were asked to draw a concept map on paper for the two scenarios presented, according to their current knowledge. An example of a conceptual map is shown in Appendix (1) After collecting the drawn conceptual map, the students were asked to research the two scenarios presented on the desired websites. The maximum duration of the search was 90 min. An example of a conceptual map is shown in Appendix (2) During the search, each student’s work page was filmed with a screen recorder. At the end of the search, they were asked to draw another conceptual map related to the two scenarios. Due to the presence of the researcher and the fact that each student had a station, it was not possible for students to copy before and after the intervention.

### Measure of learning

The tool used to collect information and measure variables was a demographic information form and a conceptual map drawn by the students. The demographic information form included age, gender, marital status, place of residence (town, village), and place of residence during education (private home, dormitory).

A concept map was used to measure learning. The concept map consists of three parts: nodes, relationships and levels. The map was drawn in such a way that the student first studied the scenario and, based on her knowledge, selected the concepts related to the scenario (nodes) and drew them in a circle or oval. In the next step, she/he used direct straight lines to show the direction of communication between concepts (relationships). The student also branched out the side concepts (levels) from the main topic according to his individual knowledge. The more major and minor connections and correctly connected branches there are in the conceptual map, the more learning has taken place. For the analysis of the concept map, 1 point is given for each meaningful correct topic (node) and 5 points for each correct added level in the map. Ten points are given for cross-connections that are not only correct but also meaningful, and 1 point is given for examples of special events or objects. The final score for the conceptual map is the sum of the scores for the subjects (nodes), the number of levels, and the number of cross-connections and examples [26]. To validate the concept map results, three professors and lecturers in the field of nursing who had complete information in the field of trauma and diabetes were used as subject experts. In addition, the grading of the final maps was reviewed and validated by an expert in the field of concept maps, who has published in this area, and by one of the professors of medical education. The conceptual map was drawn twice by the students, once before the search and once after the search. The difference between the participants’ scores before and after the search was used as a criterion to measure the amount of learning.

### Statistical analyses

Data analysis was performed using SPSS software version 2019. Means, standard deviations and frequency tables were used to describe the data. For data analysis, first the data distribution and parametric and nonparametric statistical adjustment, including paired t, Wilcoxon test, Pearson and Spearman correlation tests, were used. The significance level was considered to be less than 0.05 in all cases.

## Findings

The participants were 17 females (44%) and 21 males (56%) with a mean age of 22.44 ± 2.36 years. The paired means test was used to examine the equality of the mean scores of the conceptual map before and after the search, which showed a significant difference between the scores before and after the search in the diabetes scenario (t = 14.48, *p* < 0.001) and in the trauma scenario (t = 15.78, *p* < 0.001). (Table 1).

**Table 1:** Comparison between the mean score of the the conceptual map before and after the search according to the scenario of diabetes, trauma and total score

	Pretest	Posttest	Posttest-pretest	t(*P* value)	95% CI
	mean ± SD	mean ± SD	mean ± SD		
Diabet	18.32 ± 5.50	29.13 ± 7.54	10.81 ± 4.60	14.48(< 0.001)	(9.30,12.34)
Truama	18.58 ± 7.69	29.61 ± 7.95	11.03 ± 4.03	15.78(< 0.001)	(9.60,12.44)
Total	36.90 ± 9.66	58.74 ± 12.18	21.84 ± 7.00	19.22(< 0.001)	(19.54.24.14)

SD = Standard deviation; CI = Confidence interval

As the conceptual score is calculated from the variables number of nodes (topics), number of relationships and number of levels, the results were also analyzed by separating these variables. There was a significant difference between the number of nodes before and after the search in the diabetes scenario (t = 13.42, *p* < 0.001) and in the trauma scenario (t = 15.61, *p* < 0.001) (Table 2).

**Table 2:** Comparison between the mean number of the nodes in the conceptual map before and after the search according to the scenario of diabetes, trauma and total score

	Pretest	Posttest	Posttest-pretest	t(*P* value)	95% CI
	mean ± SD	mean ± SD	mean ± SD		
Diabet	7.84 ± 2.84	13.02 ± 4.27	5.18 ± 2.39	13.42(< 0.001)	(4.40,5.97)
Truama	7.66 ± 3.99	13.34 ± 5.20	5.69 ± 2.24	15.61(< 0.001)	(4.94,6.42)
Total	15.50 ± 5.60	26.36 ± 8.28	10.88 ± 3.72	18.00(< 0.001)	(9.64,12.09)

SD = Standard deviation; CI = Confidence interval

There was also a significant difference between the number of relationships before and after the search in the diabetes scenario (t = 12. 40, *p* < 0.001) and in the trauma scenario (t = 12.13, *p* < 0.001) (Table 3) as well as the number of levels in the diabetes scenario (z = 5.09, *p* < 0.001) and in the trauma scenario (z = 5.16, *p* < 0.001) (Table 4).

**Table 3:** Comparison between the mean number of the relationship in the conceptual map before and after the search according to the scenario of diabetes, trauma and total score

	Pretest	Posttest	Posttest-pretest	t(*P* value)	95% CI
	mean ± SD	mean ± SD	mean ± SD		
Diabet	7.00 ± 3.10	12.29 ± 4.85	5.29 ± 2.62	12.40(< 0.001)	(4.42,6.15)
Truama	6.74 ± 4.04	12.37 ± 5.70	5.63 ± 2.75	12.73(< 0.001)	(4.73,6.53)
Total	13.74 ± 5.90	24.65 ± 9.30	10.92 ± 4.55	14.78(< 0.001)	(9.42,12.42)

SD = Standard deviation; CI = Confidence interval

**Table 4:** Comparison between the mean number of the level in the conceptual map before and after the search according to the scenario of diabetes, trauma and total score

	Pretest	Posttest	Z score (*P* value)
	mean ± SD	mean ± SD	
Diabet	2.26 ± 0.60	3.37 ± 0.88	5.09(< 0.001)
Truama	2.36 ± 1.10	3.44 ± 1.08	5.16(< 0.001)
Total	4.62 ± 1.26	6.81 ± 1.57	5.29(< 0.001)

SD = Standard deviation; CI = Confidence interval

Because the mean number of the level in the conceptual map was skewed, a wilcoxon signed-ranks test was run.

The mean and standard deviation of the number of correct web pages found after searching in the diabetes and trauma scenarios were 7.08 ± 3.83 and 9.53 ± 3.89, respectively. The results showed that there was a significant correlation between the number of correct web pages searched and the learning rate in the trauma scenario (r = 0.74, *p* < 0.001) and in the diabetes scenario (r = 0.64, *p* < 0.001). There was also a significant correlation between search time and learning rate in the trauma scenario (r = 0.77, *p* < 0.001) and in the diabetes scenario (r = 0.64, *p* < 0.001) (Table 5).

**Table 5:** Correlation test between the number of Correct SERP and search time and learning rate

Variable	Pearson/Spearman correlation coefficient	*p* value
The number of correct SERP and the amount of learning rate in the trauma scenario	0.74	< 0.001
The number of correct SERP and the amount of learning rate in the diabetes scenario	0.64	< 0.001
Search time and learning rate in trauma scenario	0.77	< 0.001
Search time and learning rate in diabetes scenario	0.64	< 0.001

## Discussion

The results showed that there was a significant difference between the mean scores of the concept maps in the trauma and diabetes scenarios before and after the search, indicating learning through search. This finding is consistent with the research of Saito [27]; Zhang and Liu [21]; Liu [28]; Bhatcharya and Guizda [20]and Von Heyer [11, 29]. In addition to showing the effectiveness of search as a learning method, Liu used a conceptual map to measure learning; however, these studies only mentioned significant increases in the number of nodes, relationships, and levels before and after the intervention to determine the effectiveness of learning during search [21, 27]. They did not compare average scores. Similar to the present study, Van Heyer [29] used the average score of the four-choice test to measure the effectiveness of retrieval. Both studies emphasize the effect of search on learning, one at a high level of learning (combination and creativity with conceptual map) and the other at a low level of learning (with four-choice questions). Therefore, it can be said that search as a tool for learning can affect different levels of learning. Although Zhang believes that knowledge acquisition in the search process is superficial and occurs mainly at low levels of learning [21], the results of the present study do support the hypothesis that learning through information retrieval helps learners to use their knowledge to formulate the search and leads to exploratory learning. This type of learning creates deep insight into the subject by using high-quality information sources, which ultimately leads to high-quality learning. Based on the results of the present study and similar studies [11, 27], it can be concluded that providing tasks based on web searches can facilitate learning for learners at different cognitive levels. The preference of most students to use electronic resources, the rapid progress of science, which will be an inseparable part of universities, and the increasing number of students in medical training may cause problems for the quality of education even with the appropriate infrastructure [30]. Therefore, it is suggested that using web search as a learning tool, along with other educational methods, can go a long way toward addressing these challenges. At the level of learning details based on the conceptual map, i.e., nodes, relations and levels, the results showed that the average number of these variables in the conceptual maps drawn before and after the search increased significantly. The results are consistent with the research of Saito [27]; Zhang, Liu [21] and Liu [28]. However, in Saito et al.‘s research, the increase in the number of nodes, relationships and levels before and after the intervention was different between the two scenarios presented, as was the number of nodes, relationships and levels after the intervention in Zhang and Liu’s [21] research. It was double. The reason for the inconsistent results may be related to the type and nature of the scenarios presented in the aforementioned studies with the present study. Saito’s study is one of the scenarios about travel, which is more general, and the participants had a specific destination in mind and continued to search until they reached it, which increased the number of variables studied, i.e., nodes, relationships and levels, and the reason for this was the significant difference between the two scenarios [27]. This is although the present study was concerned with both scenarios in terms of the participants’ course and field, which had the same level of difficulty. The study by Zhang and Liu also used two general scenarios; because the participants were more familiar with the presented scenarios and their previous knowledge, they conducted a wider search and doubled the number of nodes, relationships and dimension levels compared to before the research. The generality of the topics and the knowledge that the participants already had about the tasks may be the reason why they drew maps with a wider scope in the mentioned study [21]. In general, the type of scenario used has an effect on the results at the level of learning details and their connection with the search for information. In general, general scenarios show more learning at the level of details, and specialized scenarios show a lower amount of learning; however, all of them emphasize the growth of the learning rate. It should be noted that studies have investigated the concept map as a new teaching method, and limited studies have used it for evaluation. The use of searching and drawing a concept map in detail as an evaluation method has received less attention. Therefore, it is recommended that researchers and medical scientists design studies that include scenarios with educational topics and different levels of difficulty to obtain a more accurate comparison of the effects of the search process on learning. The results showed that in both scenarios presented, there was a positive correlation between the amount of learning and the number of correct web pages searched. This finding is consistent with the study of Saito et al. [27]; Eikhoff, Teevan, White [15]; Kammerer et al. [31]. While previous studies only considered the number of pages, this study considered the number of pages searched, the correctness of the pages searched, and the information sources identified. The number of correctly searched web pages is an indicator of the effectiveness of information retrieval systems because it is related not only to the issue of access to the correct list of information sources but also to the dynamics of the search, especially the exploratory search [32]. It is suggested that regular research workshops in scientific sources be held for students every semester to remind them of reliable scientific sites and to keep students up to date. The analysis to determine the relationship between the amount of learning and the duration of the search results showed that there was a positive correlation between the search time and the amount of learning in both scenarios. The results of this part of the research are in line with previous research [11, 21]. It seems that people who spend more time searching the web and who spend more time searching between related pages have more opportunities to process information, and as the range of information related to a subject increases and more learning takes place as a result, the range of changes in their knowledge increases more [20, 31]. Rice [33] and Van Heyer [11] believe that learning while searching causes metacognition in people, and therefore, it is suggested that professors working in medical science education, especially in the field of nursing, search as a learning method along with other educational methods to increase awareness and develop high-level thinking skills to introduce to the health care provider group. Search tasks should also be considered functional learning objectives in educational topics.

The present study is the first study conducted in the field of search as a learning tool in Iran and was able to determine the effectiveness of search in the learning process of nursing students. However, it had limitations. The present semi-experimental study was conducted on a single group and only on a limited number of nursing students, so it is not possible to generalize it to other students and staff of medical sciences, and it needs further investigation with a control group. Among other limitations, the study of the amount of learning during the process of searching for information was done in one session and using a concept map. It is suggested that similar research and comparisons with other educational or assessment methods be done to check the effectiveness of this method. Additionally, the lack of studies in this area in the medical field was another limitation that made it difficult to compare and discuss properly.

## Conclusions

The results showed the effectiveness of searching for learning in nursing students. Therefore, it can be said that when people try to identify, evaluate and use information in the search process, it happens in a way of exploratory learning, which is a kind of critical thinking focused on information and causes the intellectual growth of the learner. Therefore, nursing professors can use the web and new learning and information retrieval tools as new educational tools along with other educational methods and use them in nursing education (especially scenario-based education).

### Electronic supplementary material

Below is the link to the electronic supplementary material.

Supplementary Material 1

Supplementary Material 2

## Data Availability

The datasets used during the current study are available from the corresponding author on reasonable request.
