# Supplementary material for: Information-seeking process and clinical scenario solving: introduction of a new tool in nursing education
Source: BMC Med Educ. 2023 Dec 12;23:947. doi: 10.1186/s12909-023-04943-5 (PMC10717552; doi:10.1186/s12909-023-04943-5)

Apendix 2.

Conceptual map of type 1 diabetes drawn by the participant (a) before the intervention (search)


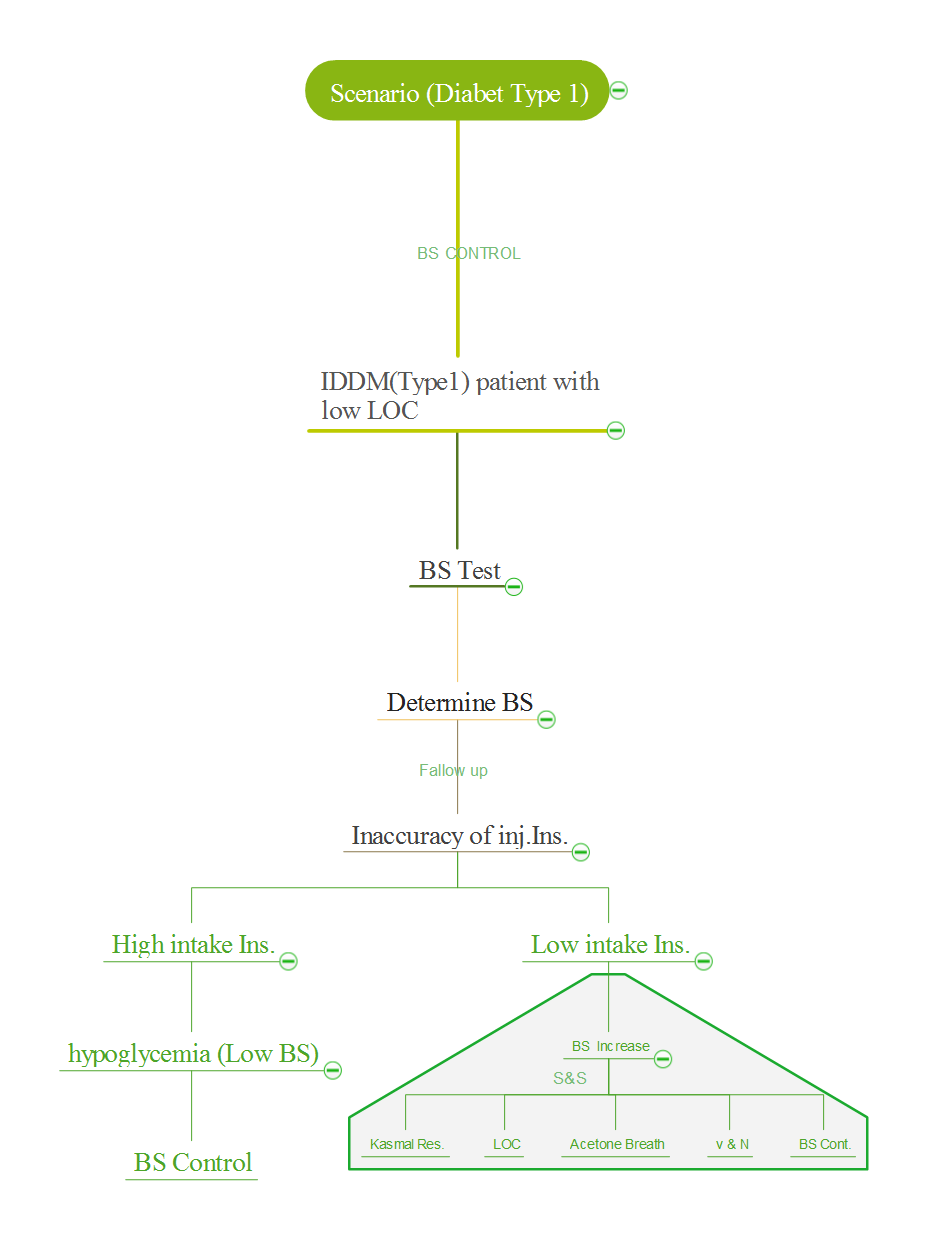


Conceptual map of type 1 diabetes drawn by the participant (a) After the intervention (search)


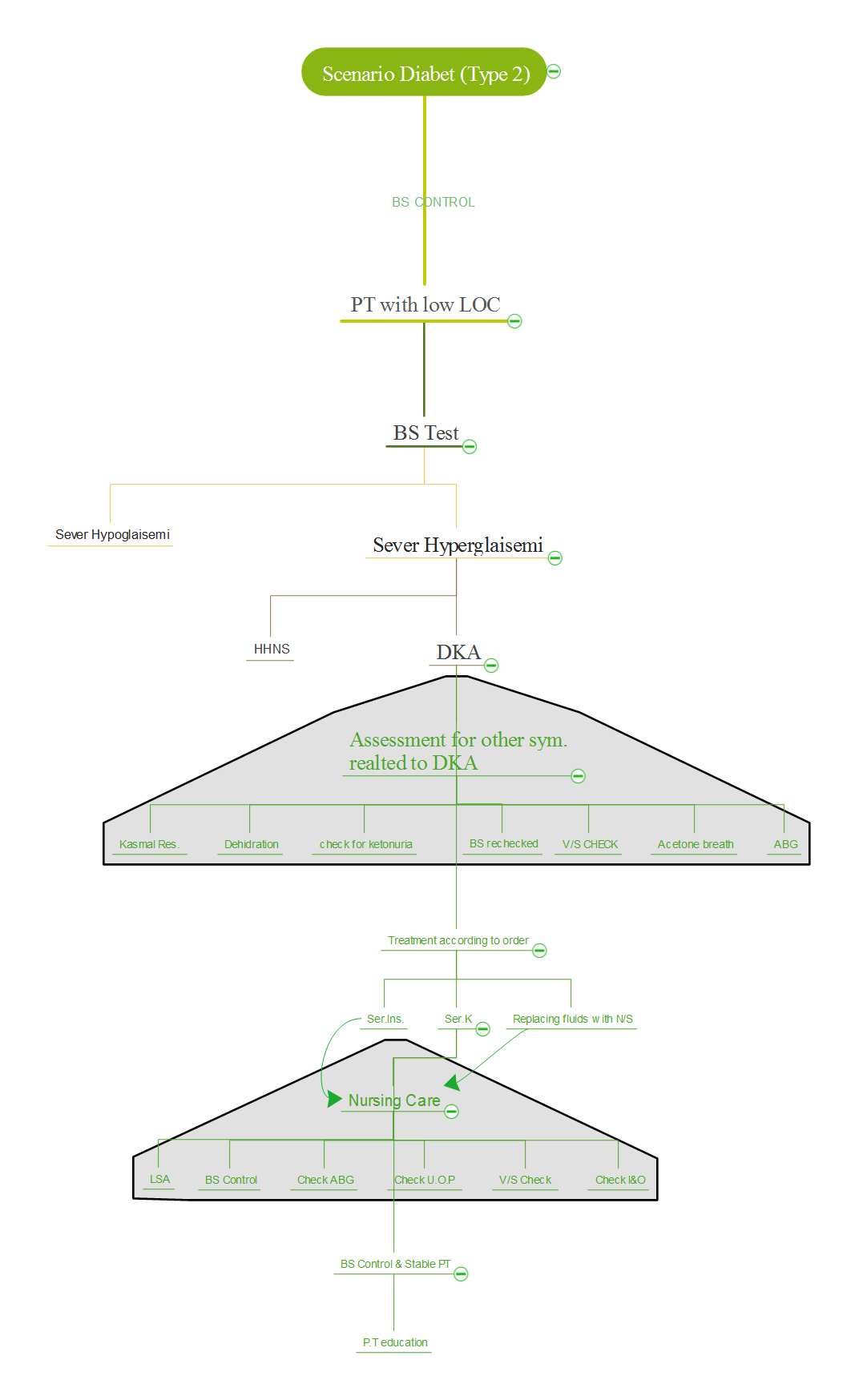

Supplement: Supplementary file 2 — Supplementary Material 2 [file 12909_2023_4943_MOESM2_ESM.docx]
